# The Influence of Urban Natural and Built Environments on Physiological and Psychological Measures of Stress—A Pilot Study

**DOI:** 10.3390/ijerph10041250

**Published:** 2013-03-26

**Authors:** Kurt Beil, Douglas Hanes

**Affiliations:** Helfgott Research Institute, National College of Natural Medicine, Portland, OR 97201, USA; E-Mail: dhanes@ncnm.edu

**Keywords:** stress, cortisol, amylase, natural environment, built environment, green space, biophilia, psychological restoration

## Abstract

Environments shape health and well-being, yet little research has investigated how different real-world environmental settings influence the well-known determinant of health known as stress. Using a cross-over experimental design; this pilot study investigated the effect of four urban environments on physiological and psychological stress measures. Participants (N = 15) were exposed on separate days to one of the four settings for 20 min. These settings were designated as Very Natural; Mostly Natural; Mostly Built and Very Built. Visitation order to the four settings was individually randomized. Salivary cortisol and alpha-amylase; as well as self-report measures of stress; were collected before and after exposure to each setting. Gender was included as a variable in analysis; and additional data about environmental self-identity, pre-existing stress, and perceived restorativeness of settings were collected as measures of covariance. Differences between environmental settings showed greater benefit from exposure to natural settings relative to built settings; as measured by pre-to-post changes in salivary amylase and self-reported stress; differences were more significant for females than for males. Inclusion of covariates in a regression analysis demonstrated significant predictive value of perceived restorativeness on these stress measures, suggesting some potential level of mediation. These data suggest that exposure to natural environments may warrant further investigation as a health promotion method for reducing stress.

## 1. Introduction

The “settings approach” to public health uses a holistic, multi-component model to describe how environments shape health and well-being [1]. This systems-based approach, established by the 1986 WHO Ottawa Charter for Health Promotion, lays the groundwork for the inclusion of healthy, supportive environments as part of the health promotion agenda [2]. The optimal design of physical environmental features is one component of this approach that contributes to a setting’s capacity to influence health [3]. The tangible infrastructure and environmental features of a place affect numerous health-determining processes. This is particularly true in urban settings, as initiatives such as the WHO/Europe’s Healthy Cities project [4] and the CDC’s Healthy Places program [5] have demonstrated. The consideration and adoption of a health-promoting approach to urban design is increasingly necessary as cities grow and the global population continues to surpass the 50% urban threshold [6]. The importance of these perspectives is reflected in the difference in prevalence of multiple physical and mental health conditions that exist between urban and rural areas [7,8,9].

One element of healthy supportive environments and urban design noted for an “upstream health promotion” capacity is the presence of trees, parks and other natural areas [10,11,12]. Epidemiological research has shown that residential proximity to these natural green spaces is associated with lower rates of morbidity and mortality in some [13,14] but not all [15] cases. Evidence suggests that one mechanism for contact with nature to positively influence health may be via their ability to facilitate stress reduction [16,17]. Exposure to natural stimuli has been shown to reduce physiological and psychological stress-related health measures in workplace environments [18,19,20], hospital settings [21,22,23] and artificial simulations [24,25]. It is hypothesized that this reaction is the result of an evolutionary adaptation known as biophilia, the “innate tendency to focus on life and life-like processes” [26]. This “psycho-evolutionary stress” (PES) theory [24] is widely regarded and many studies have supported its premise that nature has the ability to increase health and well-being by reducing stress [27].

Stress is an epidemic public health concern that negatively impacts physical and mental health, including cardiovascular, gastroenterological, immunological, neurological, endocrine and mental/ emotional health status [28,29]. The complex psychophysiological pathways of stress make measurement via one single marker impossible. Most stress research utilizes a holistic approach of collecting subjective psychological and objective physiological data to assess stress status. Psychological stress is measured via subjective rating scales. Physiological stress is often measured by salivary analysis due to the validity, reliability and ease of collection of salivary data. Salivary collection also permits simultaneous measurement of the two principal pathways of the body’s stress response: (1) the delayed-response, endocrine-mediated Hypothalamic-Pituitary-Adrenal (HPA) pathway, measured by concentration of salivary cortisol (sCort), and (2) the immediate-response, neuro-endocrine mediated Sympatho-Adreno-Medullary (SAM) pathway, measured by activity of salivary alpha-amylase (sAA) [30]. These methods have been used to measure psychological and physiological stress response after short- and long-term exposures to different environmental settings [16,31,32]. 

Few real-world experimental field studies have been conducted examining the relationship between stress and urban natural and built environmental settings. Of those that have, the vast majority utilize an initiating stressor to elevate baseline stress and facilitate measurement of stress *recovery* [24,33,34]. To the authors’ knowledge, no studies have investigated urban environments’ effect on unprovoked, *de novo* stress status. The purpose of this study was two-fold: (1) To test a method for collecting information from participants about the effects of environments on stress using a 4-arm cross-over design, and (2) To detect the differences that natural and built urban settings have on physiological and psychological measures of unprovoked, *de novo* stress. In addition, factors such as pre-existing stress, perceived restorativeness of a setting, and gender have been suggested as influential determinants of stress response to environments, and were therefore included in this pilot study. 

## 2. Methods

### 2.1. Participants

Participants were recruited from the local community via printed and internet-based methods. Anyone with a current or recent history of endocrine, neuro/psychiatric, salivary gland or acute/chronic pain disorder, or that was using certain disqualifying medications, was excluded from participating. To be eligible for enrollment, interested participants also agreed to do the following prior to each study visit: Refrain from using alcohol, tobacco and recreational drugs for at least 24 h; get a good night’s sleep; avoid strenuous activity or caffeine for 12 h; and not consume any food or liquid (except water) for one hour. Participants were given a $30 USD gift-card to a local hypermarket chain for each study visit attended, and an additional $30 gift-card if all four visits were attended (Total = $150 USD). 

A total of fifteen people (eight male, seven female) were enrolled and participated in the study. All participants completed all four study visits except for one male participant who missed one visit due to a scheduling error. Participants reported an average age of 42.3 years (range 20–61 years) and homogenous “Non-Hispanic White” racial/ethnic background. Education and income levels of the group reflected regional mean and distribution values, with a median annual income of $30,000 USD. This study was approved by the Institutional Review Board of the National College of Natural Medicine, Portland, OR USA (IRB#061912A).

### 2.2. Experimental Design

**Figure 1:**
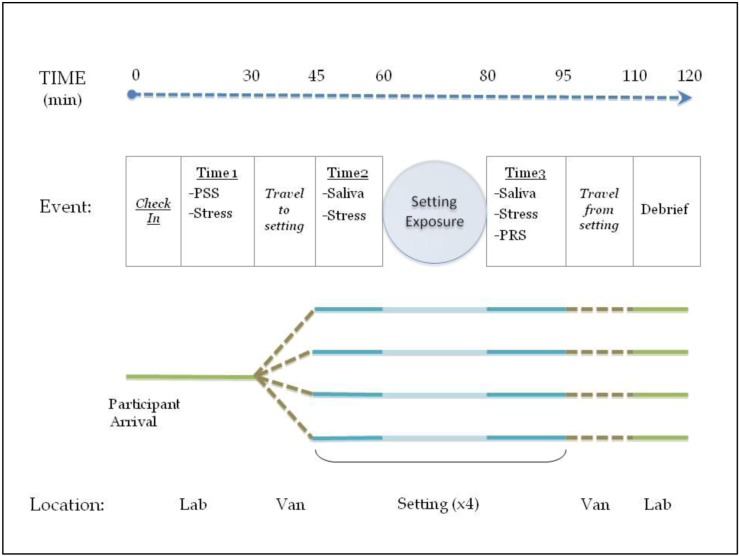
Flow diagram for each visit (×4). (PSS—Perceived Stress Scale, Stress—Subjective Stress Scale, PRS—Perceived Restorativeness Scale).

Interested participants contacted study personnel and were briefly screened for eligibility. Eligible participants reported to the study lab to sign consent forms and complete questionnaires about their health status and self-identity regarding the environment. They were asked to return for all scheduled study visits, which occurred on four separate non-consecutive weekday mornings in August 2012. The study used a four-arm cross-over design with identical visits as follows (see Figure 1): participants arrived at the study lab by 9 a.m. and were asked to turn off their cell phones and not use any electronic media or converse with other participants for the remainder of the visit. They were then asked to complete a brief health check-in form, a measure of stress experienced in the last week, and a subjective measure of current stress level (Time1). Participants were then transported in groups of three or four via passenger van to the environmental settings. Setting visitation order was individually randomized so that no participants visited the four settings in the same order. Upon arrival at the setting, participants provided a pre-exposure saliva sample and repeated the subjective stress scale (Time2). They were then instructed to sit comfortably and observe their surroundings without engaging in any activity for 20 min. After 20 min, post-exposure salivary and subjective stress data were collected (Time3). Individual on-site subjective rating scales and a focus-group debriefing back at the study lab provided information about participants’ experiences/with the individual settings. 

#### Environmental Settings

**Figure 2:**
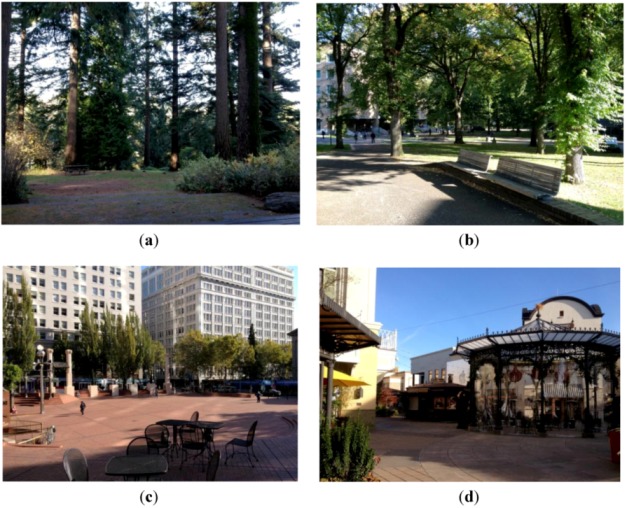
Photos depicting each of the four environmental settings experienced by participants. (**a**) Very Natural; (**b**) Mostly Natural; (**c**) Mostly Built; (**d**) Very Built.

All settings were located within 15km of the study lab, and selected on the basis of: (1) proximity to the study lab, (2) availability during the dates of the study visits, (3) presence of overhead covering to minimize sun & rain exposure, and (4) sufficient level of safety, as perceived by the study authors. The settings were categorized on a ordinal scale from “Very Natural” to “Very Built” (Figure 2(a–d)), following the method used by Matsuoka [35] as follows:

√ Very Natural: Trees, shrubs, and other natural elements with minimal evidence of human influence. Study setting was a 187-acre forested urban nature reserve
√ Mostly Natural: Presence of significant amounts of vegetation and some human influence such as walkways and buildings. Study setting was a 8.76-acre tree-lined urban park
√ Mostly Built: Majority of viewable landscape is due to human influence, with some natural elements such as trees. Study setting was a 0.92-acre urban plaza
√ Very Built: Entirety of viewable landscape is due to human influence, with minimal presence of natural elements. Study settings was a 3.46-acre outdoor shopping mall

Setting category labels were not shared with study participants at any time. Transportation to and from each setting occurred via identical rented minivans and took no longer than 15 min one-way. All settings were within 50 m of the roadway, thus minimizing the amount of walking required from setting parking areas. Visitation to the settings occurred between 9:30 and 10:30 a.m. on weekday mornings in order to minimize the presence of foot traffic and possible disruption. 

### 2.3. Measures

#### 2.3.1. Outcome Measures

##### Saliva (sCort and sAA)

Collection of saliva occurred before and after 20 min of environmental exposure at Time2 and Time3 respectively. All saliva samples were collected using Saliva Oral Swabs^®^ from Salimetrics, LLC (State College, PA, USA). Participants placed inert polymer oral collection swabs under their tongue for 2 min of passive retention before storing them in a provided swab storage tube. At the end of each study visit, samples were centrifuged for 10 min at 1,500 g, separated, and stored at −80 °C until assay. Salivary cortisol samples were analyzed in duplicate by ZRT labs (Beaverton, OR, USA) using standard ELISA. Salivary alpha-amylase was analyzed by Salimetrics LLC using a Tecan Sunrise plate reader to assess kinetic activity of 1:200 dilution at 37 °C with readings at 1 and 3 min.

##### Subjective Stress Scale (Stress)

A one-item, 0–10 rating scale was used to collect participants' perceived levels of stress, in a manner similar to that used by Nater *et al*. [36]. This instrument was administered at three times during each study visit: (1) upon initial check-in (Time1); (2) upon arrival to the environmental settings at (Time2), and (3) after 20 min of the exposure to each setting (Time3). 

#### 2.3.2. Exploratory Co-Variates (Pre-Exposure)

##### Environmental Identity (EID) Scale

The EID scale is a validated, 28-item questionnaire that measures self-identification with the natural environment and natural causes [37]. Previous research has demonstrated that EID is related to affective connection to an environment and environmental behaviors [38], but to the authors’ knowledge no studies have been conducted establishing a relationship between EID score and health status or stress response to environmental settings. 

##### Perceived Stress Scale (PSS)

The PSS is a validated 10-item self-report questionnaire that measures an individual’s response to stressful events that have occurred during a given period of time [39], in this case during the seven days prior to each study visit. To determine if pre-existing stress influenced study outcome measures, the PSS was completed during visit check-in at Time1. 

#### 2.3.3. Exploratory Co-Variates (Post-Exposure)

##### Perceived Restorativeness Scale (PRS)

The PRS is a validated 16-item scale that asks participants to rate their agreement with opinion-based statements related to environmental features [40]. It was originally developed as an instrument to test the validity of Kaplan’s neuro-cognitive model of biophilia known as Attention Restoration Theory (ART) [41], but has been used to adequately measure psychophysiological stress responses to natural and built settings according to Ulrich’s PES model [42]. It has been used to demonstrate the relationship between subjective environmental assessment and psychophysiological changes [42,43]. To account for participants’ subjective assessment of environmental settings, the PRS was completed at the conclusion of each period of exposure at Time3. 

### 2.4. Statistical Analysis

For each of the primary and secondary outcome measures, the following plan was followed: First, the effect of Visit Order on the outcome was tested to exclude it as a significant factor. Second, it was noticed that there was significant regression to the mean for almost all outcomes, so baseline values were included as covariates in all main analyses. Third, between-setting outcomes were compared using baseline values as a covariate. Where possible, mixed-model ANCOVAs were used with Setting as a within-subjects factor. Both sCort and sAA measurements were log-transformed. For some self-report outcomes, responses were distributed in a way that required non-parametric analysis, via Friedman’s test. As a follow-up to the main analysis, tests were repeated with gender included in the model to determine whether there were differences in outcomes between gender or interactions between setting and gender effects. Correlations computed for some covariates and outcome measures use all data points, including multiple measurements of individual subjects, and should therefore be considered only as descriptive measures. This is likewise true of the regression of ΔStress on PRS shown in Section 3.3.3.

## 3. Results

Initial repeated-measures analyses for all outcome measures revealed no effect of either study visit order (*i.e.*, Visit 1–4) or interaction between visit order and environmental setting. As a result, study visit order was excluded from subsequent analyses. Comparison of primary outcome measures revealed no significant correlations between sCort and sAA stress biomarkers or between these physiological measures of stress and the main psychometric stress measure (all R^2^ < 0.04). The presence of gender effects in similar studies [44,45,46] led to the decision that all outcome measure data would be analyzed by gender, subsequent to the main analyses for each measure.

### 3.1. Salivary Measures

#### 3.1.1. Cortisol (sCort)

All sCort data were subjected to a natural log transformation prior to analyses in order to normalize outcome distributions. Analyses of logCort by setting demonstrated a mean Time3 decrease in logCort relative to Time2 baseline (ΔlogCort) in all four settings, consistent with normal circadian rhythm physiology. Setting did appear to influence ΔlogCort in the hypothesized direction, *i.e.*, logCort reductions were largest after exposure to the Very Natural and Mostly Natural settings, and were larger for the Mostly Built setting than the Very Built setting. However, while these results are consistent with the PES model, ANOVA was not able to detect statistically significance ΔlogCort differences between settings (F_3,38.4_ = 0.675, *p* = 0.573). There were no gender differences detected for measurements of sCort.

#### 3.1.2. Amylase (sAA)

**Figure 3:**
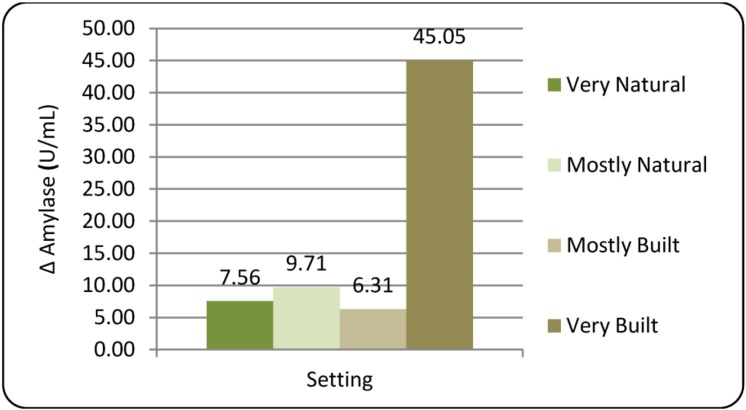
Changes in salivary amylase (∆Amylase) after 20 min exposure to environmental settings.

All sAA data was subjected to a natural log transformation prior to analysis in order to normalize outcome distributions. Analysis of sAA by setting demonstrated a mean Time3 increase relative to Time2 baseline in all four settings, though only the Very Built setting showed statistical significance for the within-group change (*p* = 0.001; See Figure 3). The elevation in sAA indicates an activation of the SAM pathway during exposure to the Very Built setting and suggests participants were highly stressed in this location at Time3 data collection. A negative correlation between ΔlogAmylase (*i.e.*, logTime3Amylase-logTime2Amylase) and logTime2Amylase (r = −0.369) was suggestive of a regression to the mean and led to inclusion of logTime2Amylase as a covariate in subsequent analyses. Repeated measures ANCOVA returned a non-significant overall effect of setting on ΔlogAmylase (F_3, 38.3_ = 1.69, *p* = 0.186). However, post hoc t-tests did show unadjusted significance in comparison of the Mostly Built and Very Built settings (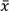 = 6.31 *vs.* 45.05 U/mL, respectively; *p* = 0.033), suggesting some difference in activation of the SAM pathway between these two built urban settings. Participant reporting during the debriefing revealed strong dislike and feelings of unease in the Very Built setting, which likely contributed to the elevation of sAA. 

Inclusion of gender in the analysis revealed that females had a mean increase in logAmylase across all four settings, while males had an overall mean decrease in logAmylase. However, ANCOVA analysis did not reveal statistical significance for the effects of either Gender (F_1,11.8_ = 3.13, *p* = 0.103) or the interaction between gender and setting (F_3,36.0_ = 0.391, *p* = 0.76). 

### 3.2. Subjective Stress Measure

**Figure 4:**
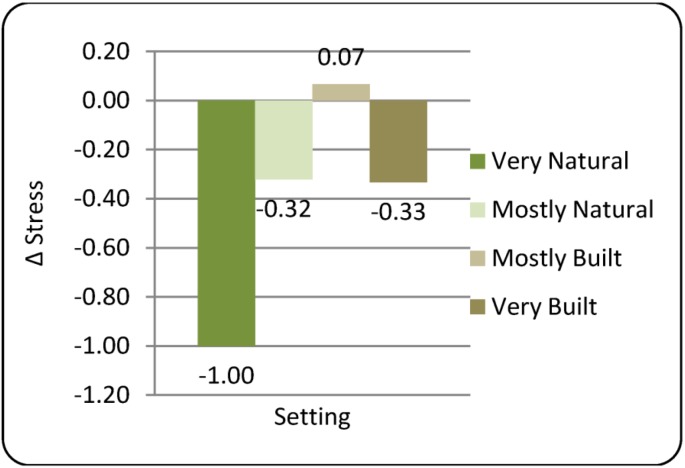
Changes in subjective stress (∆Stress) after 20 min exposure to environmental settings.

Analysis of subjective stress by setting demonstrated no between-settings difference for Time2 relative to Time1 baseline, ruling out any concern that the drive to each setting would influence subjective stress response. Conversely, setting differences were detected for stress measured at Time3 relative to Time2 (ΔStress) indicating setting exposure did have an influence on subjective stress. Larger negative ΔStress scores were detected for the natural settings compared to the built settings (Figure 4); only the Very Natural setting showed a statistically significant within-group change (*p* = 0.01 Wilcoxon signed rank). 

Comparison between settings via non-parametric Friedman’s test failed to reveal a statistically significant difference in the change in self-reported Stress (*p* = 0.140). Large negative correlations between ΔStress and Time2 Stress (r_s_ = −0.346) suggested possible regression to the mean. Inclusions of Time2 stress as a covariate was therefore used in subsequent analyses; this inclusion also yielded residual distributions suitable for parametric analysis. Parametric repeated-measures ANCOVA analysis revealed a near-statistically significant Setting main effect (F_3,40.84_ = 2.670, *p* = 0.060), after adjustment for baseline values. *Post hoc* group comparisons did demonstrate significant ΔStress differences between the Very Natural and Mostly Built settings (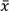 = −1.00 *vs.* +0.07, respectively; *p* = 0.008), suggesting that while these two settings did have different effects on stress status, these may have been obscured by the four-way design of this study. It is interesting to note that comments made during debriefing were mixed for the Mostly Built setting, with many participants enjoying the physical setting but disliking the noise and activity of some non-study personnel. Over-all these comments were more positive than the negative comments about the Very Built setting in which there was no statistical effect on ΔStress. Comments about the Very Natural setting were overwhelmingly positive. 

Subsequent inclusion of Gender as a factor revealed no main effect of Gender on subjective stress; however, a near-significant Setting × Gender interaction was reported (F_3,37.7_ = 2.764, *p* = 0.055). This is primarily the result of responses to the Mostly Built setting, to which only females had a positive ΔStress response (See Table 1). With gender included in the model, post hoc pair-wise comparisons revealed significant ΔStress differences between the Very Natural and both the Mostly Built (*p* = 0.003) and Very Built (*p* = 0.039) settings. 

**Table 1:** Setting × Gender adjusted means for ΔStress.

Setting	Gender	Mean ΔStress	95% Confidence Interval	
			Lower Bound	Upper Bound
Very natural	Male	−0.88	−1.73	−0.04
	Female	−1.26	−2.17	−0.34
Mostly Natural	Male	−0.33	−1.30	0.64
	Female	−0.48	−1.41	0.46
Mostly Built	Male	−0.60	−1.48	0.28
	Female	0.89	−0.07	1.84
Very Built	Male	−0.02	−0.87	0.83
	Female	−0.47	−1.39	0.45

### 3.3. Co-Variate Measures

#### 3.3.1. Environmental Identity Scale (EID)

Mean EID score for participants was 118.5 (SD = 10.1), in a possible range of 24–196 points; these results are similar to other population means [37]. Correlations were detected between EID and both ΔlogCort (r = 0.271, *p* = 0.042) and ΔlogAmylase (r = 0.278, *p* = 0.033), indicating a potential relationship between environmental identity and physiologic response. However, inclusion of EID in ANCOVA analysis did not significantly influence the effect of Setting on these salivary measures (F_3,37.9_ = 0.672, *p* = 0.575 & F_3,37.1_ = 1.672, *p* = 0.190, respectively). No correlation was detected between EID and ΔStress, indicating that environmental self-identity was unrelated to participants’ subjective experience during the study. Inclusion of EID as a covariate of ΔStress did not significantly influence the effect of Setting on subjective stress. 

#### 3.3.2. Perceived Stress Scale (PSS)

No PSS differences were detected either by setting or visit date, indicating that all groups had statistically equivalent stress levels prior to study arrival. The mean PSS score across all four study visits was 11.94 (SD = 4.96) out of a possible 40 points. This is less than the 2009 US National PSS mean of 15.84 [47], suggesting participants had lower levels of pre-existing stress at the beginning of each visit than the population average. Regarding relationship with outcome measures, PSS score was not associated with either sCort or sAA biomarker outcomes. A large correlation was detected between PSS and both Time1 (r = 0.663) and Time2 (r = 0.439) subjective stress, suggesting participants’ level of experienced stress in the previous week was related to their level of current stress during the study. However, no correlation was detected between PSS and ΔStress, suggesting that pre-existing stress was not a factor in determining changes in stress level during the experiment. Inclusion of PSS as a covariate of ΔStress in a model did however result in a near-significant Setting effect (*p* = 0.053). 

#### 3.3.3. Perceived Restorativeness Scale (PRS)

**Figure 5:**
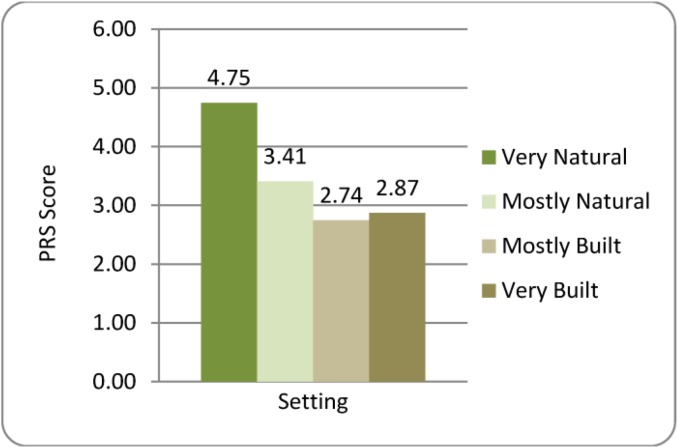
Participant’s ratings of the Perceived Restorativeness of Environmental Settings.

**Figure 6:**
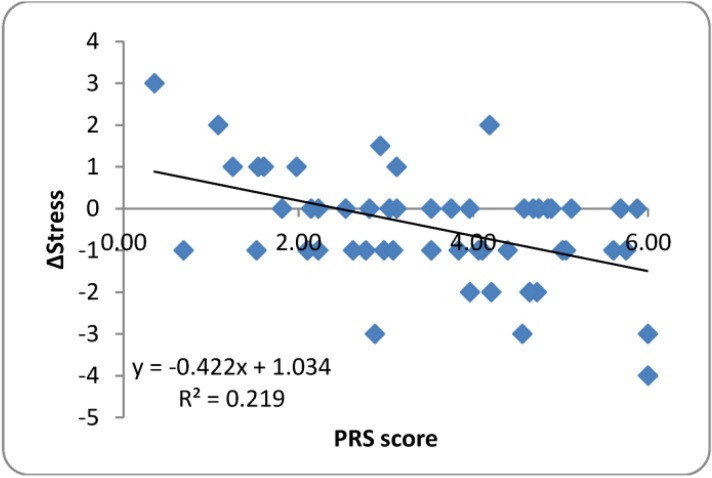
Simple linear regression between Perceived Restorativeness (PRS) score and change in Subjective Stress (ΔStress).

Highly significant setting PRS differences were found (F_3,40.97_ = 12.526, *p* < 0.001) with *post-hoc* pair-wise comparisons demonstrating that the Very Natural setting was perceived as more restorative than the other three settings (all *p* < 0.001; See Figure 5). Perceived restorativeness was not associated with either sCort or sAA biomarker outcomes, but a significant correlation was detected between PRS and ΔStress (r_s_ = −0.387). Simple linear regression to determine the effect of PRS on ΔStress produced a relationship with slope α = −0.422 (R^2^ = 0.219) demonstrating a small but reliable effect on subjective stress (See Figure 6). Inclusion of PRS as a covariate in the prior analysis of ΔStress by environmental setting showed a highly significant (*p* < 0.001) effect of PRS on ΔStress, but resulted in a highly non-significant Setting effect (F_3,41.77_ = 0.140, *p* = 0.936), suggesting that PRS may be a primary mediator of setting’s effect on subjective stress.

## 4. Discussion

Trends suggest that differences in environmental settings did influence participants’ levels of measureable stress. Salivary alpha-amylase elevation after exposure to the Very Built setting, independent of any significant change in subjective stress, identifies a physiological response that is separate from a conscious psychological component. Reductions in subjective stress after exposure to the Very Natural setting relative to the Mostly Built settings are consistent with the stress-moderating implications of PES suggested by Ulrich [24].

Data from analysis of the outcome measures was unable to support the hypotheses that natural urban settings produce more beneficial changes in measures of physiological and psychological stress relative to built urban settings. This was not unexpected considering the small sample size and low statistical power of the study. 

The low level of baseline stress among participants at Time2 (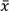 = 2.39, SD = 1.71; Scale 0–10) indicates a near-floor effect regarding baseline stress level. The likelihood of detecting measurable changes in stress, particularly after exposure to a mild and passive activity, was minimal. Therefore, the common use of an initiating, pre-exposure stressor may be warranted in future studies so that more robust physiological and psychological stress changes can be measured. 

However, the presence of a statistically significant subjective stress difference between the Very Natural and Mostly Built settings in this pilot study, despite a near-floor effect and low statistical power, does suggest a potential environmental contribution to the moderation of stress. This evidence suggests that natural environments have stress-reducing capacity beyond the restorative, therapeutic action that occurs after exposure to an acute stressor. Natural urban settings may therefore be useful for helping to create the supportive, upstream health-promotive environments that are the foundation for a more sustainable urban living experience [11,48]. Further studies will be needed to determine the strength and or “dose” of such an exposure, the duration of such effects, the effect of single *vs.* repeated exposures, and the repercussions on physical and mental health status and disease conditions. 

The gender differences in outcome measures support previous evidence suggesting women and men respond to environmental settings differently [44,45,46]. A greater decrease in subjective stress for women after Very Natural setting exposure, but greater increase after Mostly Built exposure (when men had a decrease) suggests that women may be more influenced by environmental conditions than men, in either direction of the stress scale. Comments made by female and male participants during debriefing did not demonstrate any gender differences in setting experiences, suggesting a subconscious component may be involved. Future studies in this area may want to continue including gender as a variable for analysis. 

Mean EID score consistent with other sample means indicates participants were representative of other populations regarding environmental self-identity. Correlation of EID with salivary outcome measures suggests that individuals with greater personal environmental identification may be more physiologically sensitive to their surroundings. However, this sensitivity may be generalized to all environments and not specific to the setting content as evidenced by negligible changes in ANCOVA models. Lack of correlation between EID and subjective stress markers suggests that physiologic sensitivity may occur due to sensori-perceptual level processing independent of conscious awareness. Further exploration of these mechanisms is warranted. Future studies investigating how environmental setting differences influence health outcomes may want to include an individual’s environmental self-identity in their analyses.

The mean PSS score below national average indicates a relatively relaxed sample population, though this may reflect local or regional norms (data not available). Populations with different levels of perceived baseline stress may experience different responses to setting exposures. Therefore, the generalizability of study results is limited. 

The moderately strong correlation between PRS and ΔStress suggests it is likely that restorativeness is a direct measure of a setting’s potential impact on subjective stress, regardless of a setting’s naturalness. It should be noted that a setting’s restorativeness may be independent from its categorization along a natural/built continuum [49]. As such, perceived restorativeness can differ for two settings of comparable naturalness [50,51]. For these reasons, future studies hoping to measure outcome differences between exposures to natural and built settings may want to include PRS or other subjective environmental setting measures in covariate analyses. 

It is unsurprising that there was little overlap between the salivary measures sCort and sAA. A lag of up to 18 min have been reported between the immediate-timed response of sAA and the delay-timed response of sCort after exposure to an acute stressor [52]. The limited number of salivary data collection points in this study does not allow for a full temporal correlation comparison between measures. In addition, the collection of Time3 saliva after only 20 min of setting exposure may not have been sufficient to capture the full cortisol response, given a potential 18 min delay. Lack of congruent findings between the physiological and psychological measures reflects stress response complexity, and demonstrates how a holistic approach to environmental stress research is necessary.

### Limitations

As mentioned, this pilot study was limited in its statistical power by a small sample size due to budgetary and logistical constraints. Future studies seeking to explore this area of research should consider including more participants. In addition, the recruitment of participants from the local geographic area of a mid-sized city in the Pacific Northwest of the United States limits the generalizability of this study.

Conducting an experimental field study introduces the potential for exposure to non-extraneous variables, preventing attribution of study findings to the dependant variable and making it impossible to empirically assess the validity of PES. These variables fluctuate within and between settings, as well as within and between setting visits. This variability includes both a normal range (e.g., background traffic noise of ~60 dB at the Mostly Built setting) and unforeseen outlier events (e.g., infrastructure construction noise of ~80 dB at the Very Natural setting). Participants’ comments made during debriefing shows these extraneous variables influenced conscious experience of setting exposures and directly influenced subjective stress measures. It is likely that salivary measures were also influenced by these variables [53]. A list of variables mentioned by participants includes: noise, presence of non-study personnel, past memories of setting visits, physical discomfort, air temperature, and odors. Future field studies seeking to validate environmentally-moderated stress measures should control for these factors by capturing relevant data to incorporate into data analysis models. Attempts at such exploratory data capture methods were made with the current study for the acoustic environment, but logistical issues prevented inclusion of useable data. Audio monitoring of setting decibel levels was attempted, but the equipment used was only capable of recording isolated data at prescribed time-points (*i.e.*, at Time2 and Time3). This proved to be insufficient for representing the actual experience of participants in settings with greatly fluctuating soundscapes. 

The collection of only two salivary data points provides minimal data for analysis. Collection of multiple salivary data points before and after exposure would permit incorporation of highly variable individualized daily cortisol patterns known [54,55] into data analysis while also extending the post-exposure window of extended or delayed cortisol effects, as mentioned above. 

The PRS was validated using Kaplan’s ART model, and the relationship between this type of restoration and stress has not been firmly established in the research literature. A more appropriate instrument might include assessments of the attractiveness and/or aesthetics of an environment, which are constructs used in Ulrich’s PES model. Such instruments have been used by Dijkstra *et al*., [56] and Karmanov and Hamel [49]. Use of the latter instrument may be particularly appropriate, as it includes a bipolar scale for rating the “naturalness” of a setting. The individualized data of participants’ subjective rating from this instrument would be more informative than the categorizations assigned by study personnel, and could be incorporated into co-variate analyses. It should be noted that, to the authors knowledge, neither of these instruments have been validated.

## 5. Conclusions

The purpose of this pilot study was to test a within-subjects methodology for measuring urban environmental settings’ effect on levels of stress. This information is important to understanding how environments contribute to the accumulation of stress and how this information can be used to positively affect health status in individuals and populations. 

Though this study was not able to validate the hypothesis that natural urban environments have a greater ability to positively affect unprovoked *de novo* levels of stress than built urban environments, the presence of multiple extraneous variables cannot rule-out the possibility that such an effect occur. Future studies looking to utilize an experimental field study design should control for these variables (e.g., noise, past exposures, non-study personnel, *etc*.). Consideration of environmental self-identity, perceived restorativeness and pre-existing levels of stress should be included as co-variates, and data should be analyzed by gender. Further studies are needed to determine what the effects on chronic or repeat exposures to environments might be, and if measuring the effect of these repeat exposure visits supports the epidemiological evidence. 
